# Ethnic differences in Long COVID diagnosed in primary care in England (2020–2022): an observational cohort study using OpenSAFELY

**DOI:** 10.1016/j.lanepe.2026.101605

**Published:** 2026-02-05

**Authors:** Poppy AC. Mallinson, Nick Birk, Alasdair D. Henderson, Alex Lewin, Anoop SV. Shah, Amir Mehrkar, Ben Goldacre, Giridhar R. Babu, Santosh Kumar Banjara, The OpenSAFELY collaborative, Alex J. Walker, Brian MacKenna, Peter Inglesby, Christopher T. Rentsch, Helen J. Curtis, Caroline E. Morton, Jessica Morley, Seb Bacon, George Hickman, Chris Bates, Richard Croker, David Evans, Tom Ward, Jonathan Cockburn, Simon Davy, Krishnan Bhaskaran, Anna Schultze, Elizabeth J. Williamson, William J. Hulme, Helen I. McDonald, Rosalind M. Eggo, Kevin Wing, Angel YS. Wong, Harriet Forbes, John Tazare, John Parry, Frank Hester, Sam Harper, Ian J. Douglas, Stephen JW. Evans, Liam Smeeth, Laurie A. Tomlinson, Sanjay Kinra, Rohini Mathur

**Affiliations:** a Department of Non-Communicable Disease Epidemiology, London School of Hygiene & Tropical Medicine, London, WC1E 7HT, UK; b British Heart Foundation Cardiovascular Research Centre, University of Glasgow, Glasgow, UK; c Department of Medical Statistics, London School of Hygiene & Tropical Medicine, London, WC1E 7HT, UK; d Bennett Institute for Applied Data Science, Nuffield Department of Primary Care Health Sciences, University of Oxford, OX2 6GG, UK; e Department of Population Medicine, College of Medicine, QU Health, Qatar University, Qatar; f ICMR-National Institute of Nutrition, Hyderabad, India; g Institute for Population Health Sciences, Barts and the London School of Medicine and Dentistry, Queen Mary University of London, London, UK

**Keywords:** Long COVID, Ethnicity, Primary care, Health inequalities

## Abstract

**Background:**

Long COVID continues to affect millions of adults and contribute to substantial economic burden across Europe. Ethnic inequalities in Long COVID, and the reasons underlying these, are poorly understood. We aimed to investigate ethnic differences in the incidence of diagnosed Long COVID in England using linked national primary care data.

**Methods:**

With approval from NHS England, we used linked health record data from England, 2020–2022, accessed through the OpenSAFELY platform. We applied Cox regression to compare incidence of diagnosed Long COVID in primary care across self-reported ethnicity in five groups. We explored potential explanations for these differences by 1) adjusting for sociodemographic and health-related factors, 2) restricting to those tested or hospitalised with COVID-19, 3) stratifying into 16 ethnic sub-groups.

**Findings:**

Our sample comprised 17,848,825 adults, of whom 16,970 (0.1%) had a diagnosis of Long COVID recorded in primary care. Hazard ratios (95% confidence intervals) for Long COVID compared with the white group were 1.04 (0.98–1.11) for the South Asian group, 0.84 (0.75–0.94) for the Black group, 0.97 (0.84–1.13) for the Mixed Ethnicity group, and 0.63 (0.55–0.72) for Other ethnic groups, which remained similar when adjusting for sociodemographic and health-related factors and among those tested or hospitalised for COVID-19. Disaggregating into 16 ethnic sub-groups revealed heterogeneity within groups, for example, compared with the White British group, hazard ratios were 1.21 (1.00–1.47) for the Bangladeshi group and 1.09 (0.99–1.21) for the Pakistani group, but 0.77 (0.70–0.86) for the Indian group; and 1.15 (0.95–1.40) for the Black Caribbean group but 0.61 (0.51–0.72) for the Black African group.

**Interpretation:**

Differences in Long COVID diagnoses across broad ethnic groups mask important sub-group inequalities, offering insight into underlying mechanisms and approaches to better target Long COVID services.

**Funding:**

The OpenSAFELY platform is principally funded by grants from: NHS England [2023–2025]; The Wellcome Trust (222097/Z/20/Z) [2020–2024]; MRC (MR/V015737/1) [2020–2021]. Additional contributions to OpenSAFELY and this analysis have been funded by grants from: MRC via the National Core Study programme, Longitudinal Health and Wellbeing strand (MC_PC_20030, MC_PC_20059) [2020–2022] and the Data and Connectivity strand (MC_PC_20058) [2021–2022]; NHS England via the Primary Care Medicines Analytics Unit [2021–2024]; NIHR and MRC via the CONVALESCENCE programme (COV-LT-0009, MC_PC_20051) [2021–2024] and MRC (MR/V040235/1) [2021–24].

**Research in context**

**Evidence before this study**

To our knowledge there is no existing systematic review on racial or ethnic inequalities in Long COVID in adult populations. We conducted a systematic review of PubMed updated to 20th October 2025 using the Boolean search “(differences or inequalities or inequities or disparities) and (racial or ethnic or ethnicity) and (“long COVID” or “post-acute COVID-19” or “post-COVID syndrome”)”. This returned 124 titles of which 25 studies reported racial or ethnic differences in the prevalence or incidence of Long COVID between two or more racial/ethnic groups within a country. Fourteen studies were from North or central America of which most reported higher prevalence of Long COVID among Hispanic groups than non-Hispanic White groups, while evidence for Black groups was inconsistent. Out of 11 studies from Europe, four were from Scandinavia and compared migrant versus non-migrant groups, with 3/4 reporting higher Long COVID among people with a migration background although comparisons between ethnic groups were limited by small numbers. Seven studies were from the UK, none of which had a primary aim of investigating ethnic differences in Long COVID. All reported only descriptive statistics by high-level ethnicity categories (e.g., white versus non-white, or five ethnicity categories), with inconsistent findings; 2/7 studies found no differences between ethnic groups, 2/7 reported greater risk of Long COVID in White groups, and 3/7 reported greater risk of Long COVID in certain minority ethnic groups (with no consistent pattern emerging). None of these studies investigated mechanisms or explanations for the observed ethnic differences.

**Added value of this study**

We present the first in-depth investigation of ethnic differences in diagnosed Long COVID in the UK using a large and representative primary care dataset including the largest numbers of non-white-European ethnicity individuals in Europe. Ethnic differences in diagnosed Long COVID did not align with observed ethnic differences in severe COVID-19, and did not seem to be explained by socioeconomic and health-related factors, access to testing, or hospitalisation for severe COVID-19. However, when disaggregating the broad 5-ethnicity grouping into 16 ethnic sub-groups, we noted marked differences within each broad group, which may explain the inconsistencies between previous studies. For example, compared with White British, the incidence of Long COVID was higher in the Bangladeshi group but lower in the Indian group, and higher in the Black Caribbean group but lower in the Black African group.

**Implications of all the available evidence**

These findings will help to inform the design and targeting of more inclusive Long COVID services in the UK. They also clarify inconsistencies in previous evidence and underscore the importance of disaggregating ethnic categories in health inequalities research. In the context of the broader evidence base, our results provide insights into the mechanisms underlying ethnic differences in Long COVID and highlight the context-specific nature of these inequalities.

## Introduction

Long COVID is defined as the presence of symptoms lasting more than a month after acute COVID-19 disease without another known cause.^1^ Common symptoms include fatigue, shortness of breath, difficulty concentrating and muscle pain, with up to 75% of sufferers reporting that the condition impairs their ability to carry out daily activities.^2^ Long COVID accounts for considerable ongoing health and healthcare burden across Europe, with 36 million people estimated to have been affected in the region in 2020-2023,^3^ and 3.3% of the UK population – 2 million individuals – estimated to have the condition as of early 2024.^2^ The global economic impact of Long COVID has been estimated to exceed $1 trillion per year.^4^

In the UK and many other high-income countries, the incidence of severe COVID-19 (hospitalisations and deaths due to COVID-19) during the 2020-23 pandemic was higher among minority ethnic groups than white ethnic groups, which was partially attributed to the higher socioeconomic deprivation and medical co-morbidities in these groups.^5^^,^^6^ Long COVID is known to be more common in people with co-morbidities and who were hospitalised with acute COVID-19,^7^ suggesting that ethnic disparities in the risk of Long COVID could mirror those of severe COVID-19. This would have implications for the targeting and design of Long COVID support services.^8^

There are limited data exploring ethnic disparities in Long COVID in the UK. Initial descriptive statistics from various sources suggest that disparities may not be as marked as for severe COVID-19, and that the prevalence of self-reported or diagnosed Long COVID may even be higher in white ethnic groups.^2^^,^^9^^,^^10^ There is a need to unpack these signals further and understand the mechanisms underlying them to inform planning and targeting of Long COVID services.^4^^,^^11^ We investigated ethnic differences in the incidence of diagnosed Long COVID in English primary care using a cohort study based on linked electronic health records of 17 million people in 2020–22. Our specific objectives were to: 1) Describe ethnic differences in incidence (and its changes over time) of diagnosed Long COVID in primary care in England in 2020–2022, and 2) Explore the reasons underlying these ethnics differences by 1) assessing whether they remain after adjusting for sociodemographic and health-related risk factors or restricting to people who tested positive or were hospitalised for acute COVID-19, and 2) examining their consistency across more detailed ethnic sub-groups.

## Methods

We conducted a cohort study using linked routine electronic health records of English adults accessed through OpenSAFELY (https://www.opensafely.org/). OpenSAFELY is a secure software platform for analysis of electronic health records which was established during the COVID-19 pandemic to facilitate timely and transparent use of data to inform the COVID-19 response. The data on the platform used for this analysis include primary care records of individuals registered at English General Practices (GPs) using the TPP SystmOne software system (around 40% of the English population),^12^ linked to national coronavirus testing records (Second General Surveillance System, SGSS), hospital admission records (NHS Hospital Episode Statistics, HES), and registered deaths (from the Office of National Statistics) through OpenSAFELY. All regions of England are represented, but some regions have higher usage of the TPP software (e.g., East Midlands and East).^12^ More information about OpenSAFELY is given in the Information Governance section below and at www.opensafely.org/. This study was approved by the Health Research Authority [REC reference 20/LO/0651] and by the London School of Hygiene & Tropical Medicine Ethics Board [reference 21863 and 26026]. This study is reported in accordance with the RECORD statement (https://www.record-statement.org/), the STROBE extension for studies using routinely collected data.

The study period was 1st March 2020, the month in which the COVID-19 pandemic started, to 31st August 2022, when the data was extracted. Individuals in the OpenSAFELY platform were eligible for inclusion if they were registered with a participating GP practice on 1st March 2020 and for at least one year prior to this (to ensure data on baseline covariates were available). We further excluded individuals who were missing data on age, sex or area-level deprivation (as records without these are generally missing most information and would not be included in any of the models), aged under 18 years at study start, and had a Long COVID code prior to 2020 (indicating data entry error). Participants were followed until they experienced the outcome (Long COVID diagnosis), de-registered from their practice, died or until the study end date, whichever came first. For describing incidence of diagnosed Long COVID by wave of the pandemic, we considered wave 2 to take place from 1st November 2020 to 31st March 2021, wave 3 from 1st April 2021 to 30th November 2021, and wave 4 from 1st December 2021 to 30th April 2022, based on other published work.^13^ Wave 1 was not analysed separately due to low numbers of Long COVID cases.

The primary outcome was incidence of diagnosed Long COVID, defined as presence of either one of the two long COVID diagnostic codes in primary care record after March 2020: “Ongoing symptomatic COVID-19” (symptoms lasting for 4–12 weeks, SNOMED CT code 1325181000000106) or “Post-COVID-19 syndrome” (symptoms lasting 12 weeks or more, SNOMED CT code 1325161000000102).^1^

The primary exposure was ethnicity, which was self-reported by individuals in the primary care record, then grouped into 5 high-level or 16 more detailed groups of White (White British, White Irish, Any other White background), South Asian (Indian, Pakistani, Bangladeshi, Any other South Asian background), Black (Black African, Black Caribbean, Any other Black background), Mixed (White and Asian, White and Black African, White and Black Caribbean, Any other Mixed background) and Other (Chinese, Any other). These categorisations are from the 2001 census and have previously been derived and validated in UK primary care records.^14^ We used the 5-category grouping in most analyses due to sparse data in 16 categories. We also included a category of “Unknown” ethnicity as around a fifth of individuals are missing information on ethnicity in the primary care record, which is a marker of low engagement with healthcare services implying these individuals are likely to differ from those who had ethnicity recorded.^15^ We wish to emphasise that Ethnicity is a complex and potentially dynamic social construct, with ethnic group membership defined by the individual concerned based on what is subjectively meaningful to them (rather than having a static biological basis).^16^ We are using it in this study for the purposes of monitoring inequities in the UK healthcare system, based on the assumption that it is a surrogate marker for shared exposures among people with similar social, biological, or cultural characteristics.^17^ We also note the limitations of the “Other” ethnicity category, which potentially includes individuals from heterogeneous ethnic groups (e.g., Arab, Latino/a), limiting its interpretability.

Sociodemographic and clinical covariates used in our analyses included: age and sex at start of study period; socioeconomic deprivation (proxied by index of multiple deprivation quintile of participant's neighbourhood (lower super output area) of residence at study start)^6^; body mass index based on the most recently recorded value before the study period (categorised as underweight (<18.5 kg/m^2^), normal weight (18.5–25 kg/m^2^ or 18.5–23 kg/m^2^ for Asian ethnic groups), overweight (25–30 kg/m^2^ or 23–27.5 kg/m^2^ for Asian ethnic groups), obese (30+ kg/m^2^ or 27.5+ kg/m^2^ for Asian ethnic groups) or missing); common cardiometabolic co-morbidities (hypertension, diabetes and cardiac disease history) coded at start of study period; smoking status (never, former or current; no coding of smoking status occurred in 4% of people and was assumed to indicate never smoking); household size (number of residents); care home residence (binary indicator) and number of primary care contacts made in the 12 months prior to the start of the study period, all based on previously developed codes for OpenSAFELY available on OpenCodelists (www.opencodelists.org/codelist/opensafely).

### Statistical analysis

Sociodemographic and clinical characteristics of the study population at baseline, and Long COVID diagnoses occurring during follow-up, were tabulated by ethnicity in 5 categories. We calculated incidence rates stratified by ethnicity in 5 and 16 categories by dividing the number of diagnosed Long COVID cases over the person-time of follow-up (expressed as rate per 100,000 person years). Confidence intervals for rates were calculated as rate ±1.96 ∗ standard error of the rate (computed by taking the square root of the number of events then dividing by the person-time). To explore possible drivers of ethnic disparities in Long COVID, we fitted Cox proportional hazard regression models using the start of study period as index time. We first adjusted for age (as a restricted cubic spline with 3 knots) and sex. Then we adjusted for multiple social and health-related variables that are risk factors for COVID-19 infection or severity that might account for ethnic differences in Long COVID (area-level deprivation, household size, smoking status, BMI category, co-morbidity of hypertension, diabetes and cardiac disease, number of contacts with the GP in the previous year, and care home residence). We repeated these analyses restricting to individuals who had previously tested positive for acute COVID-19 (using positive test date as the index time) or been hospitalised with acute COVID-19 as the primary reason for hospitalisation (using hospitalisation date as the index time), as we hypothesised that these sub-groups might have greater engagement with or access to health services that could lead to a Long COVID diagnosis, regardless of ethnicity. All models were stratified by geographic region (Sustainability and Transformation Partnership of the GP practice) to account for differences in baseline hazard by geographical region.

Data management was performed using Python (version 3), with analysis carried out using R (version 4.2). Code for data management and analysis, as well as codelists, are archived online (github.com/opensafely/DISECT_UK_India_COVID).

### Role of the funding source

The funders had no role in the study design, data collection, data analysis, interpretation or writing of the report.

## Results

**Fig. 1:**
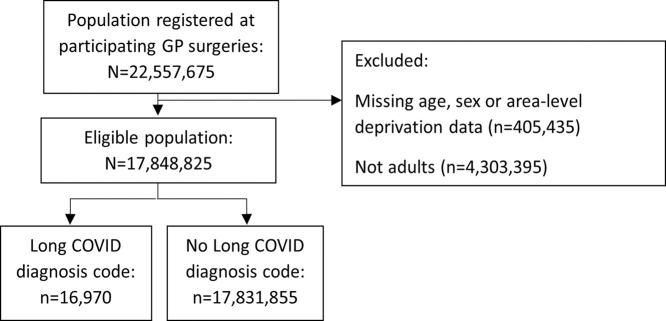
Flow diagram of study participants.

Applying the exclusion criteria to the 22,557,675 individuals registered at participating GPs in March 2020 resulted in 17,848,825 eligible adults, of whom 16,970 (0.1%) had a diagnostic code for Long COVID during the study period (2020–22) (Fig. 1).

The study population was 70% white (n = 12,566,650), 6% South Asian (n = 1,073,280), 2% Black (n = 360,300), 1% Mixed (n = 183,485), and 2% other (n = 348,820); 19% had no ethnicity recorded in primary care (n = 3,316,290) (Table 1). Compared with the White ethnic group, other groups were younger on average (e.g., mean age 52 years (standard deviation (SD) 18.3) in White group, 43 years (SD 15.4) in South Asian group, and 44 years (SD 15.6) in Black group) and more likely to live in deprived areas. Ethnic differences in the prevalence of chronic conditions such as hypertension and diabetes were varied, for example hypertension was highest in the White group (26%, n = 3,263,660), and diabetes highest in South Asian group (19%, n = 201,825). Mean number of GP contacts in the preceding year, an indicator of healthcare access, was highest in the White ethnic group (6.2, SD 9.1) and lowest in the Other ethnic groups (3.9, SD 7.3).

**Table 1:** Description of study population by ethnicity (5 categories).

Characteristic	Ethnicity in 5 categories					Unknown ethnicity	Overall
	White	South Asian	Black	Mixed	Other		
Total N (row %)	12,566,650 (70.4)	1,073,280 (6.0)	360,300 (2.0)	183,485 (1.0)	348,820 (2.0)	3,316,290 (18.6)	17,848,825 (100)
Mean age (SD)	51.6 (18.3)	42.9 (15.4)	43.9 (15.6)	39.8 (15.0)	40.4 (15.1)	47.2 (20.4)	49.8 (18.7)
Sex							
Male	6,059,090 (48.2)	560,380 (52.2)	181,160 (50.3)	89,390 (48.7)	178,430 (51.2)	1,840,820 (55.5)	8,909,270 (49.9)
Female	6,507,565 (51.8)	512,895 (47.8)	179,140 (49.7)	94,095 (51.2)	170,390 (48.8)	1,475,475 (44.5)	8,939,555 (50.1)
IMD Quintile (5 is most deprived)							
1	2,531,020 (20.1)	89,330 (8.3)	23,080 (6.4)	23,135 (12.6)	46,375 (13.3)	667,490 (20.1)	3,380,425 (18.9)
2	2,709,495 (21.2)	121,900 (11.4)	36,935 (10.3)	29,185 (15.9)	58,445 (16.8)	692,960 (20.9)	3,648,925 (20.4)
3	2,771,410 (22.1)	207,005 (19.3)	61,085 (17.0)	36,995 (20.2)	70,595 (20.2)	707,145 (21.3)	3,854,235 (21.6)
4	2,364,660 (18.8)	303,240 (28.3)	92,750 (25.7)	42,165 (23.0)	83,840 (24.0)	638,770 (19.3)	3,525,430 (19.8)
5	2,190,070 (17.4)	351,800 (32.8)	146,445 (40.6)	52,000 (28.3)	89,565 (25.7)	609,930 (18.4)	3,439,810 (19.3)
Body mass index							
Under-weight	209,285 (1.7)	27,745 (2.6)	5415 (1.5)	4175 (2.3)	12,770 (3.7)	56,225 (1.7)	315,610 (1.8)
Normal	3,642,060 (29.0)	183,975 (17.1)	85,690 (23.8)	57,280 (31.2)	114,175 (32.7)	654,735 (19.7)	4,737,915 (26.5)
Overweight	3,664,565 (29.2)	326,015 (30.4)	102,320 (28.4)	45,420 (24.8)	87,045 (25.0)	625,360 (18.9)	4,850,735 (27.2)
Obese	2,972,395 (23.7)	325,525 (30.3)	91,625 (25.4)	34,580 (18.8)	47,930 (13.7)	562,705 (17.0)	4,034,760 (22.6)
Missing	2,078,345 (16.5)	210,020 (19.6)	75,250 (20.9)	42,030 (22.9)	86,900 (24.9)	1,417,265 (42.7%)	3,909,805 (21.9)
Hypertension	3,263,660 (26.0)	206,425 (19.2)	83,500 (23.2)	25,645 (14.0)	40,810 (11.7)	698,755 (21.1)	4,318,790 (24.2)
Diabetes	1,252,530 (10.0)	201,825 (18.8)	52,355 (14.5)	16,045 (8.7)	28,740 (8.2)	279,560 (8.4)	1,831,055 (10.3)
Cardiac disease	1,110,180 (8.8)	64,915 (6.0)	14235 (4.0)	6180 (3.4)	10,835 (3.1)	249,750 (7.5)	1,456,100 (8.2)
Smoking status							
Never smoker	5,434,240 (43.2)	746,155 (69.5)	231,665 (64.3)	95,310 (51.9)	206,890 (59.3)	1,470,830 (44.4)	8,185,090 (45.9)
Former smoker	4,670,540 (37.2)	165,960 (15.5)	67,320 (18.7)	42,950 (23.4)	64,355 (18.4)	903,490 (27.2)	5,914,610 (33.1)
Current smoker	2,193,460 (17.5)	119,295 (11.1)	46,950 (13.0)	36,715 (20.0)	57,715 (16.5)	564,760 (17.0)	3,018,890 (16.9)
Mean number of GP contacts in preceding year (SD)	6.2 (9.1)	5.9 (8.7)	5.5 (8.3)	5.2 (8.7)	3.9 (7.3)	4.5 (7.2)	5.8 (8.8)
Average person-years of follow-up	2.41	2.40	2.38	2.37	2.32	2.37	2.40

Values shown are number (column %) unless specified. SD is standard deviation. Measures as of February 29th 2020 unless specified.

**Fig. 2:**
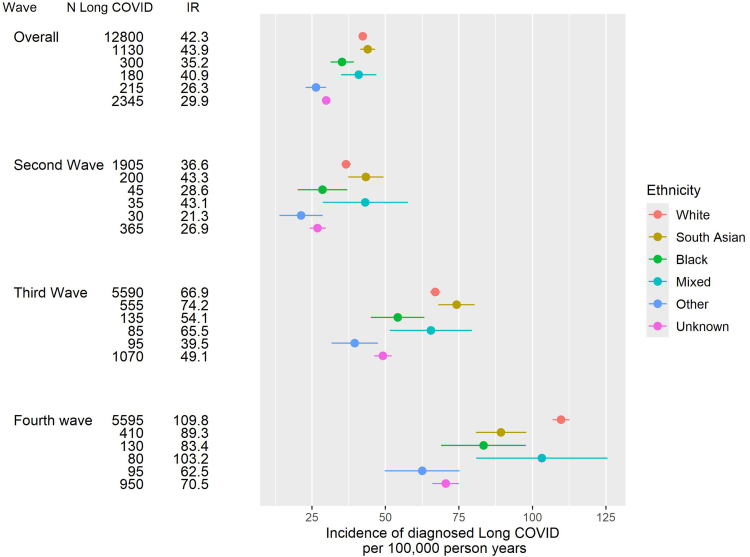
Incidence rate of diagnosed Long COVID per 100,000 person years, by ethnicity (5 categories) overall and by wave of the pandemic (OpenSAFELY 2020–22). Overall period is from 01/03/2020–31/08/2022. 2nd wave is from 01/11/2020–31/03/2021. 3rd wave is from 01/04/2021–30/11/2021. 4th wave is from 01/12/2021–30/04/2022. IR is the crude (unadjusted) incidence rate per 100,000 person years. Error bars represent 95% confidence intervals.

The average follow-up time was 2.40 years which was similar between ethnic groups. The incidence rate per 100,000 person-years (95% confidence interval) of diagnosed Long COVID over the study period was similar in White (42.3 (41.6, 43.0)) compared with South Asian (43.9 (41.4, 46.5)) and Mixed (40.9 (34.9, 46.9)) ethnic groups, and was lower in Black (35.2 (31.3, 39.2)), Other (26.3 (22.8, 29.8)) and Unknown (29.9 (28.7, 31.1)) ethnic groups (Fig. 2). Incidence rates of diagnosed Long COVID increased 2.5- to 3-fold in all ethnic groups between the 2nd and 4th waves of the pandemic. Differences by ethnicity were broadly consistent over this time, except for a greater increase in the White groups and shallower increase in the South Asian groups between the 3rd and 4th waves, which meant that by the 4th wave, incidence was lower in the South Asian than White groups (Fig. 2).

In regression models exploring whether sociodemographic or health-related factors might account for the ethnic differences observed, we noted little effect of adjustment for age and sex (Table 2). Once accounting for a wider set of social and health covariates (area-level deprivation, household size, prior GP visits, smoking status, care home residence, BMI category and co-morbidity of hypertension, diabetes and cardiac disease), the relative rate of diagnosed Long COVID became slightly lower among South Asian (hazard ratio (HR) 0.87, 95% CI 0.82, 0.93)) and Black (HR 0.74, 95% CI 0.66, 0.83) groups compared to White, while there was relatively little change amongst the Mixed and Other groups.

**Table 2:** Incidence rates and hazard ratios for diagnosed Long COVID in primary care in 5 ethnicity categories, unadjusted, adjusted for age and sex, and adjusted for health and socioeconomic factors.

Ethnicity	N	n Long COVID cases	Long COVID incidence rate per 100,000 PY (95% CI)	Hazard ratio for Long COVID (95% CI)		
				Unadjusted	Age and sex adjusted	Multivariable adjusted^a^
White	12,566,650	12,800	42.3 (41.6, 43.0)	1 (ref)	1 (ref)	1 (ref)
South Asian	1,073,280	1130	43.9 (41.4, 46.5)	1.04 (0.98, 1.11)	0.96 (0.90, 1.03)	0.87 (0.82, 0.93)
Black	360,300	300	35.2 (31.3, 39.2)	0.84 (0.75, 0.94)	0.79 (0.71, 0.89)	0.74 (0.66, 0.83)
Mixed	183,485	180	40.9 (34.9, 46.9)	0.97 (0.84, 1.13)	0.96 (0.83, 1.11)	0.96 (0.83, 1.11)
Other	348,820	215	26.3 (22.8, 29.8)	0.63 (0.55, 0.72)	0.65 (0.56, 0.74)	0.68 (0.60, 0.78)
Unknown	3,316,290	2340	29.9 (28.7, 31.1)	0.71 (0.68, 0.74)	0.80 (0.76, 0.84)	0.88 (0.84, 0.92)

PY, person years; CI, confidence interval.

a Adjusted for area-level deprivation, household size, GP visits, smoking status, care home residence, BMI category and co-morbidity of hypertension, diabetes and cardiac disease.

We examined the same associations among the subset of people who had been recorded as having a positive COVID-19 test at least 4 weeks before their Long COVID diagnosis and among those who were hospitalised with acute COVID-19 (Table 3), as we hypothesised that these individuals might be more similar in terms of healthcare access and follow-up regardless of ethnicity, indicating if observed ethnic differences were due to healthcare seeking behaviour. The results of these analyses were broadly similar to the overall findings, except notably that the incidence of diagnosed Long COVID among South Asian groups post-hospitalisation was lower relative to White groups. After adjustment for health and sociodemographic factors, the ethnic differences were attenuated (or even reversed) in the post-hospitalisation group, although low numbers of cases for this analysis suggests these results should be interpreted cautiously.

**Table 3:** Hazard Ratios for diagnosed Long COVID by ethnicity in 5 categories following a) positive COVID-19 test, and b) hospitalisation due to COVID-19.

Ethnicity	a) HR (95% CI) for Long COVID following a positive COVID-19 test				b) HR (95% CI) for Long COVID following hospitalisation with COVID-19			
	N	n Long COVID cases	Age-sex adjusted	Multivariable adjusted^a^	N	n Long COVID cases	Age-sex adjusted	Multivariable adjusted^a^
White	2,246,075	5325	1 (ref)	1 (ref)	67,050	890	1 (ref)	1 (ref)
South Asian	210,615	515	0.94 (0.85, 1.03)	0.72 (0.65, 0.79)	9620	125	0.70 (0.58, 0.85)	1.56 (1.04, 2.35)
Black	64,205	115	0.77 (0.64, 0.92)	0.66 (0.55, 0.80)	3230	40	0.72 (0.52, 0.99)	0.99 (0.53, 1.85)
Mixed	35,725	70	0.97 (0.76, 1.22)	0.90 (0.71, 1.13)	1070	15	0.69 (0.40, 1.19)	1.28 (0.52, 3.15)
Other	41,810	75	0.82 (0.65, 1.02)	0.76 (0.61, 0.96)	1790	35	1.08 (0.76, 1.54)	1.98 (1.08, 3.63)
Unknown	591,135	990	0.86 (0.81, 0.92)	0.90 (0.84, 0.96)	15,220	175	0.88 (0.75, 1.04)	0.98 (0.65, 1.49)

HR, hazard ratio; CI, confidence interval.

a Adjusted for area-level deprivation, household size, GP visits, smoking status, care home residence, BMI category and co-morbidity of hypertension, diabetes and cardiac disease.

When stratifying incidence rates by 16 ethnic groups, substantial heterogeneity within each of the high-level 5 groups was revealed (Table 4). For example, relative to the White British group, there was higher incidence in the Pakistani, Bangladeshi and Caribbean groups, and lower incidence in the Other White, Indian, Other Asian, Black African, Other Black, and Chinese groups, although estimates for some of these were imprecise to due to small numbers.

**Table 4:** Incidence of diagnosed Long COVID by ethnicity in 16 categories (OpenSAFELY 2020–22).

Ethnicity (5 categories)	Ethnicity (16 categories)	N	n Long COVID cases	Crude incidence of diagnosed Long COVID	Age-sex adjusted HR (95% CI) compared to White British
White	White British	11,056,620	11765	44.1	1 (ref)
	White Irish	90,355	90	41.9	1.03 (0.84, 1.27)
	Other White	1,419,675	950	28.3	0.64 (0.60, 0.69)
South Asian	Indian	452,800	395	36.5	0.77 (0.70, 0.86)
	Pakistani	311,860	430	56.9	1.09 (0.99, 1.21)
	Bangladeshi	73,280	105	58.7	1.21 (0.99, 1.47)
	Other Asian	235,335	200	36.1	0.78 (0.68, 0.90)
Black	Caribbean	84,445	105	51.7	1.15 (0.95, 1.40)
	African	193,710	135	29.2	0.61 (0.51, 0.72)
	Other Black	82,145	65	32.5	0.69 (0.54, 0.88)
Mixed	White and Caribbean	43,235	55	51.2	1.13 (0.86, 1.48)
	White and African	34,475	30	38.0	0.80 (0.56, 1.14)
	White and Asian	37,265	35	37.4	0.82 (0.58, 1.15)
	Other mixed	68,505	60	37.8	0.85 (0.66, 1.10)
Other	Chinese	110,990	25	10.4	0.26 (0.17, 0.38)
	Other ethnic groups	237,830	185	33.3	0.75 (0.65, 0.86)
Unknown	Unknown ethnicity	3,316,290	2345	29.9	0.71 (0.68, 0.74)

HR, hazard ratio; CI, confidence interval.

## Discussion

In this large primary care population in England, the incidence of diagnosed Long COVID was similar among White, South Asian and Mixed ethnic groups, but lower among Black and Other ethnic groups. These ethnic differences were not accounted for by adjusting for differences in sociodemographic or health-related characteristics, and were similar in the subpopulation that was hospitalised with acute COVID-19 prior to their Long COVID diagnosis. We present the first data to our knowledge on Long COVID disaggregated into 16 ethnic sub-groups, which revealed marked heterogeneity within the broader 5-ethnicity categorisation. Compared to the White British group, the incidence of diagnosed Long COVID was higher in Pakistani, Bangladeshi and Caribbean groups, and lower in Other White, Indian, Black African and Chinese groups.

Published data describing ethnic differences in diagnosed Long COVID in UK primary care are based on three data sources: the TPP GP database system, which covers ∼34% of GPs in the UK, used in our study and two previous descriptive studies^9^^,^^10^; the EMIS GP database system, which covers ∼56% of GPs and has a different geographical distribution than TPP^18^; and the National Primary Care Sentinel Cohort (PCSC), a representative network of 743 (12%) practices in England.^19^^,^^20^ Analysis of the EMIS data (up to April 2021) found that, compared with the White groups, incidence of diagnosed Long COVID (based on any Long COVID related code) was higher in South Asian (rate ratio (RR) 1.50) and Black (RR 1.15) groups, and lower in the Mixed (RR 0.92) and Other (RR 0.62) groups.^18^ However, a high proportion of the study sample had “unknown” ethnicity (46% of Long COVID cases), and this group had the highest incidence of diagnosed Long COVID (RR 1.78), indicating the results may be strongly influenced by missing data mechanisms. Other reasons for these different findings could be the time period covered (corresponding roughly to wave 2 in our analysis), and different geographical coverage of the two datasets (TPP being over-represented in the East and Northeast of England and EMIS being over-represented in London and the South East). Given that the socioeconomic, health and migration backgrounds of ethnic groups in the UK tend to vary regionally, our findings may not be so applicable to areas such as London. Analysis of the PCSC data (also up to April 2021) found that, compared with the White groups, prevalence of diagnosed Long COVID was lower in the Asian groups (prevalence ratio (PR) 0.87) and Other groups (PR 0.91), and higher in the Black groups (PR 1.14), although comparison with our study is difficult because they used a different ethnicity categorisation (Asian rather than South Asian) and restricted to people with a positive test for COVID-19 (5% of the total sample), which could have markedly affected their results.^19^ Nevertheless, it is notable that both these datasets observed slightly higher rates of diagnosed Long COVID in Black groups compared with White groups, in contrast to our findings.

Data on self-reported Long COVID from population-based surveys has the advantage of including all cases of perceived Long COVID, rather than being restricted to those who seek care and get diagnosed at the GP. However, surveys are often limited by lower sample sizes, particularly for certain minority ethnic groups. The nationally representative COVID-19 Infection Surveys conducted regularly by the Office for National Statistics reported a lower prevalence of self-reported Long COVID in Asian groups compared with White groups, which was statistically robust in all survey periods between 2020 and 2022 (prevalence ratios between 0.57 and 0.83). Among Black and Mixed groups, the prevalence of self-reported Long COVID was lower compared with White groups from April 2021 onwards, but similar prior to this point (although confidence intervals were wide throughout due to low numbers).^21^ Another study of self-reported Long COVID using 7 pooled longitudinal cohort studies reported a prevalence ratio of 0.80 (95% CI 0.54–1.19) for Non-White compared with White groups, but did not disaggregate further due to low numbers.^9^ Together this evidence suggests that ethnic differences in Long COVID are not as consistent and marked as have been observed for acute COVID-19 hospitalisations and mortality, where minority ethnic groups faced a consistent excess risk.^6^

Another aim of our analysis was to understand the reasons underlying ethnic differences in Long COVID in the UK. There is limited published literature with which to directly compare our findings, as the above studies were limited to simple descriptives. One study using another GP database, CRPD Aurum, analysed the incidence of at least one symptom of Long COVID at least 12 months after acute COVID-19 (rather than diagnostic coding for Long COVID).^22^ They found that compared with the White group, the rate was lower in the Asian group, but similar in the Black, Mixed and Other groups, although after adjusting for socio-demographics and pre-COVID symptoms, the rates increased in all groups relative to the white groups. It is possible that certain minority ethnic groups experience more symptoms associated with Long COVID but that this does not translate to diagnosis or self-report of Long COVID, for example due to less knowledge of the condition or differential symptom presentation.^23^ Higher burden of Long COVID-related symptoms, but not necessarily diagnosis, among ethnic minority groups, has been noted in the United States.^24^^,^^25^

We are not aware of any other studies from the UK that have examined ethnic differences disaggregated beyond the 5-group categorisation of White, Black, Asian/South Asian, Mixed or Other. The marked heterogeneity that we noted within these groups (often in qualitatively different directions) suggests the 5-group categorisation may not be useful for understanding ethnic differences in Long COVID in the UK. Similarly in Denmark, a study of electronic health records noted variation within broader ethnic groups; for example, rates of Long COVID were higher among people of Iraqi and Turkish origin, but not among people of Pakistani or Afghan origin, compared with native Danes.^26^ In contrast, among people hospitalised for acute COVID-19 in the Netherlands and invited to attend a post-COVID clinic, Long COVID symptoms were more common in Surinamese, Moroccan and Turkish (but not “Other”) ethnic groups compared with Dutch individuals.^27^

For other health outcomes, such as mortality and morbidity, there is heterogeneity within each of the broad 5-category ethnic groups, but not usually in qualitatively different directions as we have observed for Long COVID. For example, age-standardised all-cause mortality in the UK is lower in all ethnic sub-groups compared with the white group, but the Black Caribbean and Bangladeshi populations face the highest mortality within the Black and Asian groups, respectively.^28^^,^^29^ For severe acute COVID-19, all minority ethnic groups experienced increased age-standardised rates of hospitalisation and death compared with white groups, although again the magnitude of this excess risk varied between subgroups, with for example higher rates in Black African populations than other Black groups.^6^ Similarly, in the national Understanding Society study, all minority ethnic groups reported worse self-rated health and physical functioning than the white group, but this was most pronounced among Pakistani and Bangladeshi participants.^30^ Examining ethnic disparities across more disaggregated ethnic subgroups is clearly valuable and informative across a range of health outcomes, but appears particularly critical for Long COVID where using 5 ethnic categories only could mask ethnic differences completely.

It is likely that the mechanisms underlying ethnic differences in Long COVID are variable and context-specific. Inconsistent findings from previous studies reporting only the 5-category ethnic groupings may at least in part be due to varying composition of these broad ethnic groupings in different areas of the UK, highlighting a need to disaggregate these heterogeneous groups further. The finding from a number of data sources that incidence of Long COVID was among the highest in White groups was surprising because we know that on average White groups had lower incidence of severe acute COVID-19 than most minority ethnic groups.^6^ Underdiagnosis of Long COVID in primary care, as well as under-reporting of self-reported Long COVID in population-based surveys, are likely to be greater in minority ethnic groups.^23^ Qualitative studies in people with Long COVID from minority ethnic backgrounds in the UK reveal several reasons why diagnosis and reporting might be lower in these communities, including a lack of awareness of Long COVID in their communities, fear of stigma and disbelief in their symptoms from healthcare providers and/or community members, challenges in navigating complex referral pathways, and other structural health system factors including language barriers.^31^^,^^32^ A study in a predominantly Pakistani community reflected that “experiences of mistrust and fear were rooted in the disproportionate impact of COVID-19 on ethnic minorities”, in turn leading to reduced care seeking for Long COVID.^32^ In the Netherlands, minority ethnic (Turkish and Moroccan) individuals with Long COVID reported facing numerous barriers to accessing primary care, including language, digital literacy and stereotyping by healthcare professionals.^33^ Within the more detailed 16-category ethnic grouping, higher rates of diagnosed Long COVID in Black Caribbean, Pakistani and Bangladeshi groups could reflect the higher rates of severe COVID-19 and other co-morbidities, which all increase the risk of Long COVID.^6^ We note that although the strength of evidence comparing these groups to the white group was only moderate due to low numbers, there is strong evidence of heterogeneity within each 5-category group, and strong evidence for an elevated risk would be observed if Bangladeshi and Pakistani groups (which share a similar history and certain aspects of culture) were combined. It is less clear why other ethnic groups, such as Indian, Black African, Chinese and Other White, had markedly lower incidence of diagnosed Long COVID than the White British group. These groups comprise a higher proportion of people born outside the UK (over two-thirds for Black African, Other White and Chinese^34^), who experience greater barriers to accessing primary care than people born in the UK, especially during the COVID-19 pandemic.^35^ Lower levels of acculturation in these groups could further reduce awareness of Long COVID and affect how symptoms manifest, making it harder for individuals or healthcare providers to identify the condition.^23^ Another contributing explanation could be the so-called “healthy migrant effect”, wherein more favourable health profiles are observed among (economic) migrants than native populations due to selection of who is able to migrate,^36^ although direct evidence for such selection effects is limited.^37^^,^^38^ Specific explanations could also apply in certain groups, for example the low incidence of acute COVID-19 among the Chinese group likely partly accounts for the particularly low incidence of diagnosed Long COVID in this group.^6^

We have used a large, real-world dataset to explore differences in diagnosis of Long COVID among ethnic groups in the UK, exploring reasons for these differences and disaggregating into more detailed ethnic groupings for the first time. However, some limitations are noted. Our study can only draw conclusions about diagnosed Long COVID as coded in primary care. Yet, surveys suggest that the majority of people in the UK who report experiencing Long COVID do not have a formal diagnosis.^2^ It is reassuring that the nationally representative ONS Long COVID surveys (which do not restrict to diagnosed cases) are broadly consistent with our findings, although data are not available for disaggregated ethnic groupings due to small sample size. Coding of Long COVID in primary care data systems (using SNOMED codes) only became widely available in November 2020. We started our study in March 2020 to align with the timelines of the pandemic, and because diagnoses can be backdated. However, rates of diagnosed Long COVID before 2021 are likely to be severely undercounted; we present ethnic differences by wave of the pandemic for this reason. Ethnicity was unknown for 19% of the study population. Ethnicity recording is a marker of engagement with healthcare services, so a lack of ethnicity data in primary care records may be indicative of lower attendance as well as poorer health outcomes (or conversely, may also indicate healthy individuals who rarely avail primary care services). Previous studies using the same database have used models to predict missing ethnicity and noted this had little effect on the findings.^6^ Individuals who died due to severe acute COVID-19 are unable to progress to Long COVID, which could have introduced selection bias as most minority ethnic groups experienced higher age-specific COVID-19 mortality than the white population.^6^ However, the rate of death due to COVID-19 was low in absolute terms (<0.1% in all ethnic groups in first two waves), and age, the key underlying factor in COVID-19 mortality, is not a strong risk factor for Long COVID. Together this suggests that although our observed associations may be slightly attenuated, our conclusions are unlikely to be substantially biased by mortality selection. Finally, the GP database we used (TPP) is not fully representative of England (e.g., London, Southeast and Northwest England are under-represented) and our findings may be less applicable to these areas.

To understand ethnic differences in diagnosed Long COVID, it is necessary to examine disaggregated ethnic groupings beyond the broad 5-ethnicity categorisation that has been used in research reports to date. Large-scale data such as electronic health records, despite limitations, will be necessary to facilitate these insights, for example, through comparison of symptomology (and its time trends) in diagnosed and undiagnosed individuals, care pathways (including referral, diagnosis, treatment), effectiveness of interventions, and long-term impacts, across disaggregated ethnic groupings. Our data suggest that although ethnic differences in Long COVID are not as marked as for severe COVID-19, there may be higher rates of disease in certain historically disadvantaged minority groups (e.g., Bangladeshi, Pakistani and Black Caribbean), emphasising the need for Long COVID services to be sensitive to these communities. Reasons underlying the lower rates of diagnosed Long COVID in other minority ethnic groups (e.g., Indian, Black African) are unclear and future research should aim to identify these. Our findings highlight the role of social and health system (rather than genetic) factors underlying ethnic disparities in Long COVID. Given the continued scale of the Long COVID crisis in the UK, further research is needed to inform the provision of effective and equitable Long COVID care services.

## Contributors

Author contributions were as follows: Conceptualisation (RM, NB, SK, PACM), data curation (NB, BG, AM, OSC), formal analysis (NB), funding acquisition (SK, RM, LT, AL, ASVS, GRB, SKB, BG, OSC), methodology (PACM, NB, AL, LT, SK, RM), project administration (BG, AM, OSC, LT, RM), software (BG, OSC), supervision (RM), visualisation (PACM), writing – original draft (PACM), writing – review and editing (all). For this study, NB and PACM verified the underlying data. All authors reviewed and approved the final manuscript. PACM had final responsibility for the decision to submit for publication.

## Data sharing statement

All data were linked, stored and analysed securely using the OpenSAFELY platform, https://www.opensafely.org/, as part of the NHS England OpenSAFELY COVID-19 service. Data include pseudonymised data such as coded diagnoses, medications and physiological parameters. No free text data are included. All code is shared openly for review and re-use under MIT open license (github.com/opensafely/DISECT_UK_India_COVID). Detailed pseudonymised patient data is potentially re-identifiable and therefore not shared.

## Declaration of interests

LAT holds an NIHR Professorship (NIHR302405), reports grants from MRC, Wellcome, NIHR in the past 3 years, has received travel expenses for MHRA expert advisory group meetings, and is an unpaid member of 3 trial steering committees (NIHR funded). BG has received research funding from the Bennett Foundation, the Laura and John Arnold Foundation, the NHS National Institute for Health Research (NIHR), the NIHR School of Primary Care Research, NHS England, the NIHR Oxford Biomedical Research Centre, the Mohn-Westlake Foundation, NIHR Applied Research Collaboration Oxford and Thames Valley, the Wellcome Trust, the Good Thinking Foundation, Health Data Research UK, the Health Foundation, the World Health Organisation, UKRI MRC, Asthma UK, the British Lung Foundation, and the Longitudinal Health and Wellbeing strand of the National Core Studies programme; he has previously been a Non-Executive Director at NHS Digital, and written a review for SofS in UK Department of Health; he also receives personal income from speaking and writing for lay audiences on the misuse of science. AM is a senior clinical researcher at the University of Oxford in the Bennett Institute, which is funded by contracts and grants obtained from the Bennett Foundation, Wellcome Trust, NIHR Oxford Biomedical Research Centre, NIHR Applied Research Collaboration Oxford and Thames Valley, Mohn-Westlake Foundation, and NHS England; he has represented the RCGP in the health informatics group and the Profession Advisory Group that advises on access to GP Data for Pandemic, and is a former employee and interim Chief Medical Officer of NHS Digital (now merged into NHS England), having left NHS Digital in January 2020. ADH reported receiving payments from Bayer for travel and consultancy outside the submitted work. All other authors declare no conflicts of interest.

## References

[1] Venkatesan P. (2021). NICE guideline on long COVID. Lancet Respir Med.

[2] Self-reported coronavirus (COVID-19) infections and associated symptoms, England and Scotland - Office for National Statistics. https://www.ons.gov.uk/peoplepopulationandcommunity/healthandsocialcare/conditionsanddiseases/articles/selfreportedcoronaviruscovid19infectionsandassociatedsymptomsenglandandscotland/november2023tomarch2024.

[3] Statement – 36 million people across the European Region may have developed long COVID over the first 3 years of the pandemic. https://www.who.int/europe/news/item/27-06-2023-statement---36-million-people-across-the-european-region-may-have-developed-long-covid-over-the-first-3-years-of-the-pandemic.

[4] Al-Aly Z., Davis H., McCorkell L. (2024). Long COVID science, research and policy. Nat Med.

[5] Irizar P., Pan D., Kapadia D. (2023). Ethnic inequalities in COVID-19 infection, hospitalisation, intensive care admission, and death: a global systematic review and meta-analysis of over 200 million study participants. eClinicalMedicine.

[6] Mathur R., Rentsch C.T., Morton C.E. (2021). Ethnic differences in SARS-CoV-2 infection and COVID-19-related hospitalisation, intensive care unit admission, and death in 17 million adults in England: an observational cohort study using the OpenSAFELY platform. Lancet Lond Engl.

[7] Tsampasian V., Elghazaly H., Chattopadhyay R. (2023). Risk factors associated with post-COVID-19 condition: a systematic review and meta-analysis. JAMA Intern Med.

[8] Norredam M., Hayward S., Deal A., Agyemang C., Hargreaves S. (2022). Understanding and addressing long-COVID among migrants and ethnic minorities in Europe. Lancet Reg Health Eur.

[9] Thompson E.J., Williams D.M., Walker A.J. (2022). Long COVID burden and risk factors in 10 UK longitudinal studies and electronic health records. Nat Commun.

[10] Henderson A.D., Butler-Cole B.F., Tazare J. (2024). Clinical coding of long COVID in primary care 2020-2023 in a cohort of 19 million adults: an OpenSAFELY analysis. eClinicalMedicine.

[11] Knuppel A., Boyd A., Macleod J., Chaturvedi N., Williams D.M. (2024). The long COVID evidence gap in England. Lancet.

[12] Andrews C., Schultze A., Curtis H. (2022). OpenSAFELY: representativeness of electronic health record platform OpenSAFELY-TPP data compared to the population of England. Wellcome Open Res.

[13] Nab L., Parker E.P.K., Andrews C.D. (2023). Changes in COVID-19-related mortality across key demographic and clinical subgroups in England from 2020 to 2022: a retrospective cohort study using the OpenSAFELY platform. Lancet Public Health.

[14] Mathur R., Bhaskaran K., Chaturvedi N. (2014). Completeness and usability of ethnicity data in UK-based primary care and hospital databases. J Public Health (Oxf).

[15] Mathur R. (2015).

[16] Ethnicity harmonised standard – government analysis function. https://analysisfunction.civilservice.gov.uk/policy-store/ethnicity-harmonised-standard/#presentation-england-and-wales.

[17] Lee C. (2009). ‘Race’ and ‘ethnicity’ in biomedical research: how do scientists construct and explain differences in health?. Soc Sci Med.

[18] Walker A.J., MacKenna B., Inglesby P. (2021). Clinical coding of long COVID in English primary care: a federated analysis of 58 million patient records in situ using OpenSAFELY. Br J Gen Pract.

[19] Mayor N., Meza-Torres B., Okusi C. (2022). Developing a long COVID phenotype for postacute COVID-19 in a national primary care sentinel cohort: observational retrospective database analysis. JMIR Public Health Surveill.

[20] Meza-Torres B., Delanerolle G., Okusi C. (2022). Differences in clinical presentation with long COVID after community and hospital infection and associations with all-cause mortality: English sentinel network database study. JMIR Public Health Surveill.

[21] Prevalence of ongoing symptoms following coronavirus (COVID-19) infection in the UK statistical bulletins - Office for National Statistics. https://www.ons.gov.uk/peoplepopulationandcommunity/healthandsocialcare/conditionsanddiseases/bulletins/prevalenceofongoingsymptomsfollowingcoronaviruscovid19infectionintheuk/previousReleases.

[22] Subramanian A., Nirantharakumar K., Hughes S. (2022). Symptoms and risk factors for long COVID in non-hospitalized adults. Nat Med.

[23] Khunti K., Banerjee A., Evans R.A., Calvert M. (2024). Long COVID research in minority ethnic populations may be lost in translation. Nat Med.

[24] Khullar D., Zhang Y., Zang C. (2023). Racial/ethnic disparities in post-acute sequelae of SARS-CoV-2 infection in New York: an EHR-based cohort study from the RECOVER program. J Gen Intern Med.

[25] Song Z., Giuriato M. (2023). Demographic and clinical factors associated with long COVID. Health Aff Proj Hope.

[26] Mkoma G.F., Agyemang C., Benfield T. (2024). Risk of long COVID and associated symptoms after acute SARS-COV-2 infection in ethnic minorities: a nationwide register-linked cohort study in Denmark. PLoS Med.

[27] Chilunga F.P., Appelman B., van Vugt M. (2023). Differences in incidence, nature of symptoms, and duration of long COVID among hospitalised migrant and non-migrant patients in the Netherlands: a retrospective cohort study. Lancet Reg Health Eur.

[28] Age standardised mortality rates for England and Wales by sex and ethnic group - Office for National Statistics. https://www.ons.gov.uk/peoplepopulationandcommunity/birthsdeathsandmarriages/lifeexpectancies/datasets/agestandardisedmortalityratesforenglandandwalesbysexandethnicgroup.

[29] Inequalities in mortality involving common physical health conditions, England - Office for National Statistics. https://www.ons.gov.uk/peoplepopulationandcommunity/healthandsocialcare/healthinequalities/bulletins/inequalitiesinmortalityinvolvingcommonphysicalhealthconditionsengland/21march2021to31january2023.

[30] Evandrou M., Falkingham J., Feng Z., Vlachantoni A. (2016). Ethnic inequalities in limiting health and self-reported health in later life revisited. J Epidemiol Community Health.

[31] Smyth N., Ridge D., Kingstone T. (2024). People from ethnic minorities seeking help for long COVID: a qualitative study. Br J Gen Pract.

[32] Baz S.A., Fang C., Carpentieri J.D., Sheard L. (2022). ‘I don't know what to do or where to go’. Experiences of accessing healthcare support from the perspectives of people living with long COVID and healthcare professionals: a qualitative study in Bradford, UK. Health Expect.

[33] Nyaaba G.N., Torensma M., Goldschmidt M.I. (2025). Experiences of stigma and access to care among long COVID patients: a qualitative study in a multi-ethnic population in the Netherlands. BMJ Open.

[34] (2018). People born outside the UK. https://www.ethnicity-facts-figures.service.gov.uk/uk-population-by-ethnicity/demographics/people-born-outside-the-uk/latest/#place-of-birth-uk-or-non-uk-by-ethnicity.

[35] Zhang C.X., Boukari Y., Pathak N. (2022). Migrants' primary care utilisation before and during the COVID-19 pandemic in England: an interrupted time series analysis. Lancet Reg Health Eur.

[36] Stanaway F.F., Khalatbari-Soltani S., Zhu L. (2025). Inequalities in all-cause mortality by ethnicity in the United Kingdom: a systematic review and meta-analysis. Lancet Reg Health Eur.

[37] Hiam L., Zhang C.X., Burns R., Darlington-Pollock F., Wallace M., McKee M. (2022). What can the UK learn from the impact of migrant populations on national life expectancy?. J Public Health Oxf Engl.

[38] Agyemang C. (2019). Comfy zone hypotheses in migrant health research: time for a paradigm shift. Public Health.

