# Supplementary material for: Ethnic differences in Long COVID diagnosed in primary care in England (2020–2022): an observational cohort study using OpenSAFELY
Source: Lancet Reg Health Eur. 2026 Feb 5;63:101605. doi: 10.1016/j.lanepe.2026.101605 (PMC12906119; doi:10.1016/j.lanepe.2026.101605)
Supplement: Appendix [file mmc1.docx]

Appendix Supplementary Material – Further information about the Open Safely collaborative group and information governance, ethics and patient involvement in OpenSafely.

List of members of the OpenSafely Collaborative:

The OpenSafely Collaborative members (excluding those in the study author list)

| First Name | Surname | Affiliation |
| --- | --- | --- |
| Alex J | Walker | Bennett Institute for Applied Data Science, University of Oxford, Oxford, UK |
| Brian | MacKenna | Bennett Institute for Applied Data Science, University of Oxford, Oxford, UK |
| Peter | Inglesby | Bennett Institute for Applied Data Science, University of Oxford, Oxford, UK |
| Christopher T | Rentsch | London School of Hygiene & Tropical Medicine |
| Helen J | Curtis | Bennett Institute for Applied Data Science, University of Oxford, Oxford, UK |
| Caroline E | Morton | London School of Hygiene & Tropical Medicine |
| Jessica | Morley | Bennett Institute for Applied Data Science, University of Oxford, Oxford, UK |
| Seb | Bacon | Bennett Institute for Applied Data Science, University of Oxford, Oxford, UK |
| George | Hickman | Bennett Institute for Applied Data Science, University of Oxford, Oxford, UK |
| Chris | Bates | TPP, Leeds, UK |
| Richard | Croker | Bennett Institute for Applied Data Science, University of Oxford, Oxford, UK |
| David | Evans | Bennett Institute for Applied Data Science, University of Oxford, Oxford, UK |
| Tom | Ward | Bennett Institute for Applied Data Science, University of Oxford, Oxford, UK |
| Jonathan | Cockburn | TPP, Leeds, UK |
| Simon | Davy | Bennett Institute for Applied Data Science, University of Oxford, Oxford, UK |
| Krishnan | Bhaskaran | London School of Hygiene & Tropical Medicine, UK |
| Anna | Schultze | London School of Hygiene & Tropical Medicine, UK |
| Elizabeth J | Williamson | London School of Hygiene & Tropical Medicine, UK |
| William J | Hulme | Bennett Institute for Applied Data Science, University of Oxford, Oxford, UK |
| Helen I | McDonald | London School of Hygiene & Tropical Medicine, UK |
| Rosalind M | Eggo | London School of Hygiene & Tropical Medicine, UK |
| Kevin | Wing | London School of Hygiene & Tropical Medicine, UK |
| Angel Y S | Wong | London School of Hygiene & Tropical Medicine, UK |
| Harriet | Forbes | London School of Hygiene & Tropical Medicine, UK |
| John | Tazare | London School of Hygiene & Tropical Medicine, UK |
| John | Parry | TPP, Leeds, UK |
| Frank | Hester | TPP, Leeds, UK |
| Sam | Harper | TPP, Leeds, UK |
| Ian J | Douglas | London School of Hygiene & Tropical Medicine, UK |
| Stephen J W | Evans | London School of Hygiene & Tropical Medicine, UK |
| Liam | Smeeth | London School of Hygiene & Tropical Medicine, UK |

Information governance and ethical approval

NHS England is the data controller of the NHS England OpenSAFELY COVID-19 Service; TPP is the data processor; all study authors using OpenSAFELY have the approval of NHS England. This implementation of OpenSAFELY is hosted within the TPP environment which is accredited to the ISO 27001 information security standard and is NHS Information Governance Toolkit compliant.

Patient data has been pseudonymised for analysis and linkage using industry standard cryptographic hashing techniques; all pseudonymised datasets transmitted for linkage onto OpenSAFELY are encrypted; access to the NHS England OpenSAFELY COVID-19 service is via a virtual private network (VPN) connection; the researchers hold contracts with NHS England and only access the platform to initiate database queries and statistical models; all database activity is logged (<https://jobs.opensafely.org/>); only aggregate statistical outputs leave the platform environment following best practice for anonymisation of results such as statistical disclosure control for low cell counts.

The service adheres to the obligations of the UK General Data Protection Regulation (UK GDPR) and the Data Protection Act 2018. The service previously operated under notices initially issued in February 2020 by the Secretary of State under Regulation 3(4) of the Health Service (Control of Patient Information) Regulations 2002 (COPI Regulations), which required organisations to process confidential patient information for COVID-19 purposes; this set aside the requirement for patient consent. As of 1 July 2023, the Secretary of State has requested that NHS England continue to operate the Service under the COVID-19 Directions 2020. In some cases of data sharing, the common law duty of confidence is met using, for example, patient consent or support from the Health Research Authority Confidentiality Advisory Group.

Taken together, these provide the legal bases to link patient datasets using the service. GP practices, which provide access to the primary care data, are required to share relevant health information to support the public health response to the pandemic, and have been informed of how the service operates.

Patient and Public Involvement and Engagement (PPIE)

OpenSAFELY has involved patients and the public in various ways: we developed a public website that provides a detailed description of the platform in language suitable for a lay audience (https://opensafely.org); we have participated in two citizen juries exploring public trust in OpenSAFELY; we have co-developed an explainer video (https://www.opensafely.org/about/); we have patient representation who are experts by experience on our OpenSAFELY Oversight Board; we have partnered with Understanding Patient Data to produce lay explainers on the importance of large datasets for research; we have presented at various online public engagement events to key communities (e.g., Healthcare Excellence Through Technology; Faculty of Clinical Informatics annual conference; NHS Assembly; HDRUK symposium); and more. To ensure the patient voice is represented, we are working closely to decide on language choices with appropriate medical research charities (e.g., Association of Medical Research Charities). We will share information and interpretation of our findings through press releases, social media channels, and plain language summaries.
